# Supplementary material for: Taking advantage of reference-guided assembly in a slowly-evolving lineage: Application to Testudo graeca
Source: PLoS One. 2024 Aug 9;19(8):e0303408. doi: 10.1371/journal.pone.0303408 (PMC11315351; doi:10.1371/journal.pone.0303408)

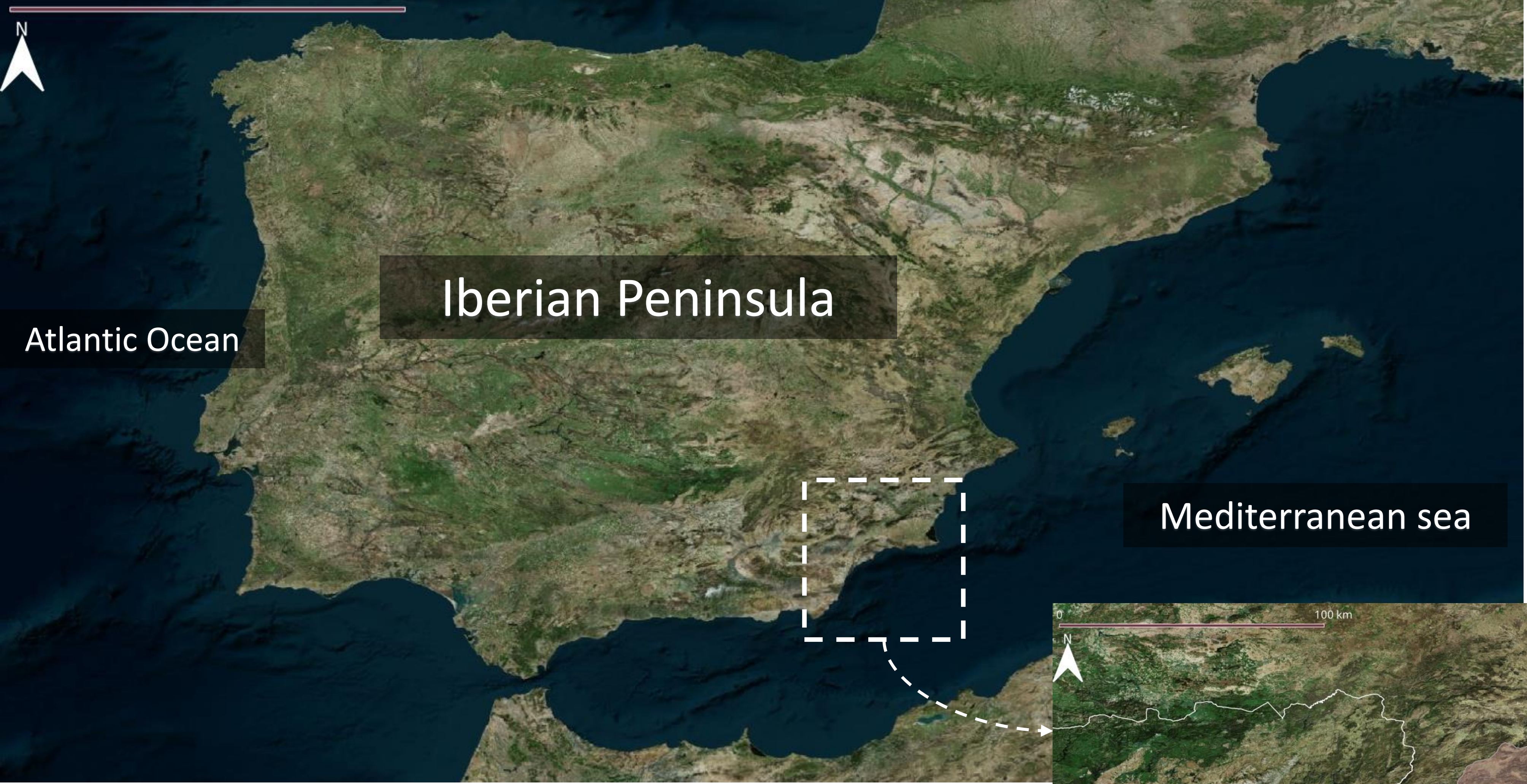

**UTM**		**Decimal degree** (WGS84)		**Degrees Minutes Seconds** (WGS84)	
East	West	Latitude	Longitude	Latitude	Longitude
622500	4150500	37.49330197	-1.61425692	-37º -29' -35.887" S	1º 36' 51.325" W

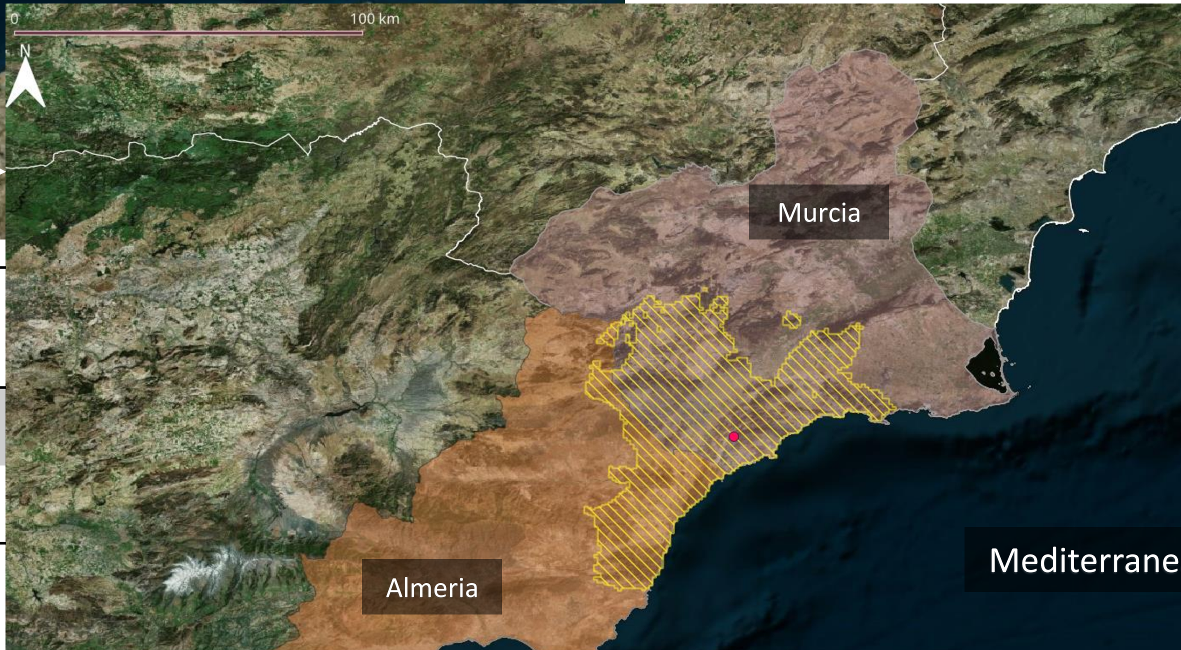

Supplement: S1 Map — (PDF) [file pone.0303408.s001.pdf]
