# Supplementary material for: Taking advantage of reference-guided assembly in a slowly-evolving lineage: Application to Testudo graeca
Source: PLoS One. 2024 Aug 9;19(8):e0303408. doi: 10.1371/journal.pone.0303408 (PMC11315351; doi:10.1371/journal.pone.0303408)
Supplement: S1 Appendix — (PDF) [file pone.0303408.s002.pdf]

[FastQC Report of R1]

[FastQC Report of R2]

References

Wingett SW, Andrews S. Fastq screen: A tool for multi-genome mapping and quality control. F1000Res. 2018;7:1338.
