# Supplementary material for: Taking advantage of reference-guided assembly in a slowly-evolving lineage: Application to Testudo graeca
Source: PLoS One. 2024 Aug 9;19(8):e0303408. doi: 10.1371/journal.pone.0303408 (PMC11315351; doi:10.1371/journal.pone.0303408)
Supplement: S1 Fig — Upper row: summary statistics (scaffold, right, and contig L50, left) for SOAPdenovo2 assemblies using a range of k-mer size before scaffolding with ntJoin. The red dot indicates the assembly retained for the next step (scaffolding with ntJoin). Lower row: Percentage of gaps remaining after scaffolding the k = 87 SOAPdenovo2 assembly with ntJoin for k-mer size (right) and word size (left). The red dot indicates the assembly retained for the next step (GapCloser). (PDF) [file pone.0303408.s003.pdf]

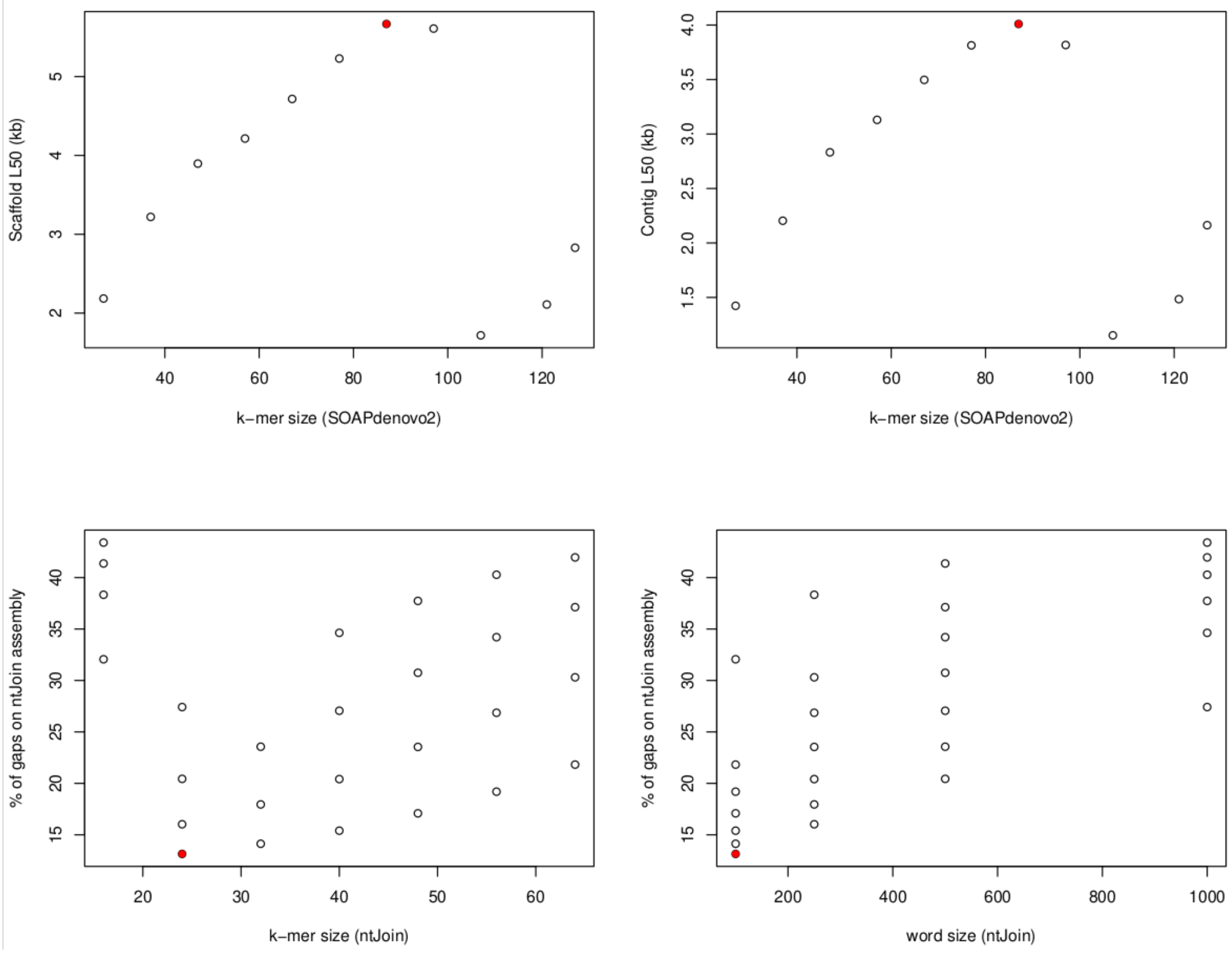

S3 Figure 1

Upper row: summary statistics (scaffold, right, and contig L50, left) for SOAPdenovo2 assemblies using a range of k-mer size before scaffolding with ntJoin. The red dot indicates the assembly retained for the next step (scaffolding with ntJoin).

Lower row: Percentage of gaps remaining after scaffolding the k=87 SOAPdenovo2 assembly with ntJoin for k-mer size (right) and word size (left). The red dot indicates the assembly retained for the next step (GapCloser).
