# Supplementary material for: Taking advantage of reference-guided assembly in a slowly-evolving lineage: Application to Testudo graeca
Source: PLoS One. 2024 Aug 9;19(8):e0303408. doi: 10.1371/journal.pone.0303408 (PMC11315351; doi:10.1371/journal.pone.0303408)
Supplement: S2 Fig — Kimura divergence of each repetitive element copy from its consensus is displayed as a barplot. (PDF) [file pone.0303408.s004.pdf]

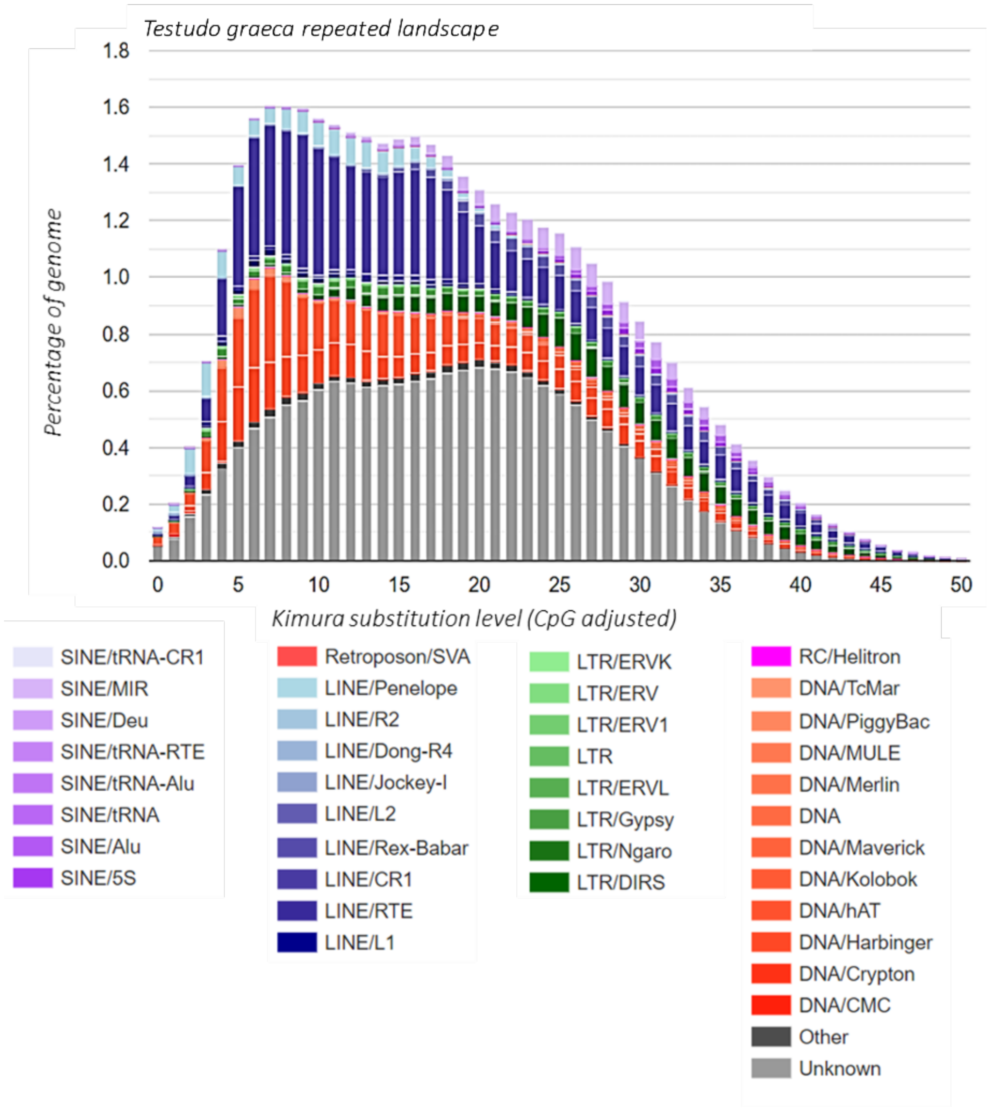

S4 Figure 1

Kimura divergence of each repetitive element copy from its consensus is displayed as a barplot.
