# Supplementary material for: Taking advantage of reference-guided assembly in a slowly-evolving lineage: Application to Testudo graeca
Source: PLoS One. 2024 Aug 9;19(8):e0303408. doi: 10.1371/journal.pone.0303408 (PMC11315351; doi:10.1371/journal.pone.0303408)
Supplement: S3 Fig — Phylogeny of full-length Testudo graeca mitochondrial sequences using T. marginata as an outgroup. Bootstrap support is indicated at nodes. (PDF) [file pone.0303408.s005.pdf]

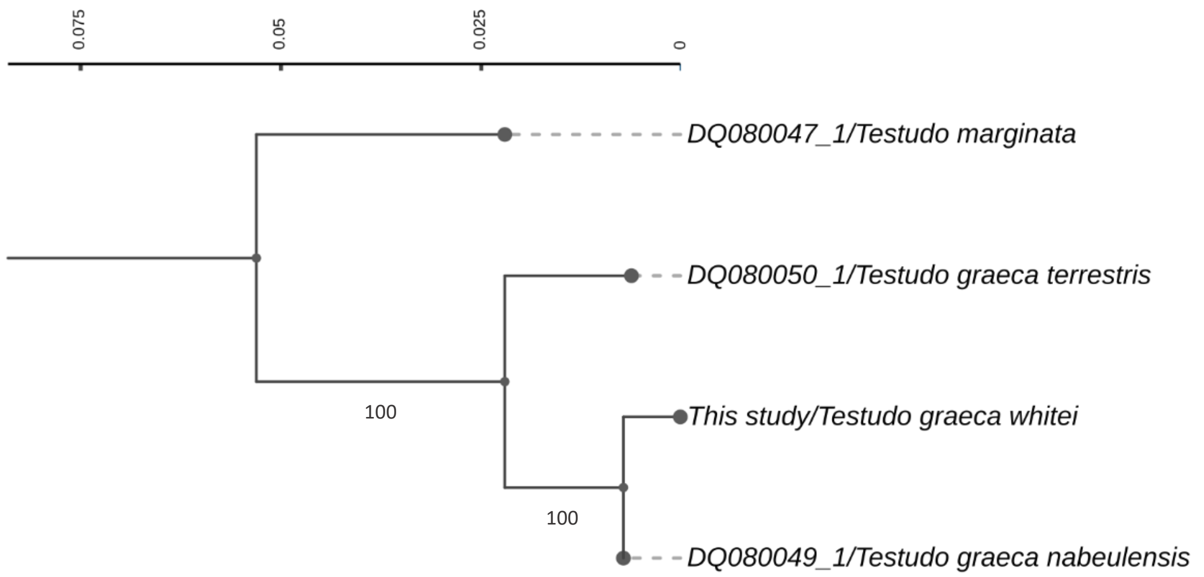

S5 Figure 1

Phylogeny of full-length *T. graeca* mitochondrial sequences. Bootstrap support is indicated at nodes.

References:

Letunic I., Bork P., Interactive Tree of Life (iTOL) v6: recent updates to the phylogenetic tree display and annotation tool, Nucleic Acids Research, 2024; https://doi.org/10.1093/nar/gkae268
